# Dr. Dwight G. Hubbard

**Published:** 1914-09

**Authors:** 


Dr. Dwight G. Hubbard, Buffalo, 1869, died in Buffalo, July 25.

